# Efficient Generation of Virus-Free iPS Cells Using Liposomal Magnetofection

**DOI:** 10.1371/journal.pone.0045812

**Published:** 2012-09-25

**Authors:** Hyo Young Park, Eun Hyung Noh, Hyung-Min Chung, Man-Jong Kang, Eun Young Kim, Se Pill Park

**Affiliations:** 1 Miraebio Research Institute, Mirae Biotech, Seoul, Korea; 2 Jeju National University Stem Cell Research Center, Jeju National University, Jeju, Korea; 3 CHA Stem Cell Institute and CHA Biotech, Pochon CHA University, Seoul, Korea; 4 Department of Animal Science and Technology, College of Agriculture and Life Science, Chonnam National University, Gwangju, Korea; 5 Faculty of Biotechnology, College of Applied Life Sciences, Jeju National University, Jeju, Korea; Universidad de Castilla-La Mancha, Spain

## Abstract

The generation of induced pluripotent stem (iPS) cells is a powerful tool in regenerative medicine, and advances in nanotechnology clearly have great potential to enhance stem cell research. Here, we introduce a liposomal magnetofection (LMF) method for iPS cell generation. Efficient conditions for generating virus-free iPS cells from mouse embryonic fibroblast (MEF) cells were determined through the use of different concentrations of CombiMag nanoparticle-DNA(pCX-OKS-2A and pCX-cMyc)-lipoplexes and either one or two cycles of the LMF procedure. The cells were prepared in a short reprogramming time period (≤8 days, 0.032–0.040%). Among the seven LMF-iPS cell lines examined, two were confirmed to be integration-free, and an integration-free LMF-iPS cell line was produced under the least toxic conditions (single LMF cycle with a half-dose of plasmid). This cell line also displayed *in vitro*/*in vivo* pluripotency, including teratoma formation and chimeric mouse production. In addition, the safety of CombiMag-DNA lipoplexes for the transfection of MEF cells was confirmed through lactate dehydrogenase activity assay and transmission electron microscopy. These results demonstrated that the LMF method is simple, effective, and safe. LMF may represent a superior technique for the generation of virus-free or integration-free iPS cell lines that could lead to enhanced stem cell therapy in the future.

## Introduction

Induced pluripotent stem (iPS) cells resemble embryonic stem (ES) cells in morphology, gene expression, epigenetic status, and *in vitro* differentiation [1], [2]. Like ES cells, iPS cells have potential as therapies, as disease models, or in drug screening. iPS cells have clear advantages: they can be made from adult cells, avoiding the controversial need for a human embryo, and they can be derived from people with diseases to create models or even therapies based on a specific individual’s genetic make-up. Since the initial generation of iPS cells by a pioneer group [1], a number of results have been achieved using a variety of species, cell types, and vectors [3]–[6]. However, common to all of these modalities is: (1) the necessity of expression of four defined transcription factors, Oct3/4, Klf4, Sox2, and c-Myc, for the successful generation of iPS cells, and (2) the need for resolution of the problem of oncogenesis and insertional mutagenesis caused by viral vector systems (retrovirus [7], [8], lentivirus [3], [9], or inducible lentivirus [10], [11]) for stable therapeutic application of iPS cells. Consequently, attention has been focused on non-integrating vector systems. Three categories of non-integrating systems have been developed: excisable (piggyBac transposon [12] and Floxed lentivirus [13], [14]), non-integrating (adenovirus [15] and plasmid [16]), and DNA-free (protein [17], [18] and mRNA [19]). While the excisable vector system yields a higher reprogramming efficiency (>100-fold) than other non-viral systems, laborious screening of excised lines and examination of non-specific genetic alteration is inevitably required before and after transfection. Conversely, virus-free or DNA-free delivery systems present a safe reprogramming option for making iPS cells, but the efficiency is extremely low and the generation time is very long. An ideal iPS cell generation method for clinical application would consider both of the most important characteristics, safety and efficiency. Recently, nanotechnologies have shown great potential to enhance stem-cell research and stem-cell–based therapeutics. Such methods could be useful in measuring, understanding, and manipulating stem cells [20]. As a universal method enhancing non-viral gene delivery, magnetofection (MF) can be an efficient and reliable method for the introduction of foreign DNA into target cells. According to our previous patent (KR1020070064784), MF led to significantly (three-fold) higher gene delivery in ES cells compared with lipid-based transfection. In the case of iPS cell generation, we expect that the efficiency of non-viral gene delivery can be increased by MF using nanoparticles or polyplexes.

In the present study, we introduce liposomal magnetofection (LMF) for iPS cell generation. This method, in which ternary complexes of cationic lipids self-assemble with plasmid DNA associated with superparamagnetic iron oxide nanoparticles, potentiates gene transfection by applying a magnetic field to concentrate CombiMag-DNA lipoplexes (manufactured by Chemicell, Berlin, Germany) onto target cells. We optimized the safer and more effective LMF method in making virus-free iPS cells from mouse embryonic fibroblast (MEF) cells. Different concentrations of two plasmids (pCX-OKS-2A and pCX-cMyc) with CombiMag were tested, and one vs. two cycles of LMF was compared. Using four treatment groups, simple and efficient conditions were optimized for the generation of LMF-iPS cells with very short reprogramming times. Among seven LMF-iPS cell lines selected from these four treatment groups, two were confirmed to be integration-free. This result demonstrated that a stable, integration-free LMF-iPS cell line was produced under the least toxic conditions (a single LMF procedure and a half-dose of plasmid), and that this cell line had *in vitro* and *in vivo* pluripotency similar to that of other cell lines. In addition, we analyzed the safety of CombiMag-DNA lipoplexes in the generation of LMF-iPS cells using a non-radioactive cytotoxicity assay and transmission electron microscopy (TEM).

## Results

### Generation of Mouse iPS Cells using pCX-OKS-2A and pCX–cMyc Plasmids and LMF

**Figure 1:**
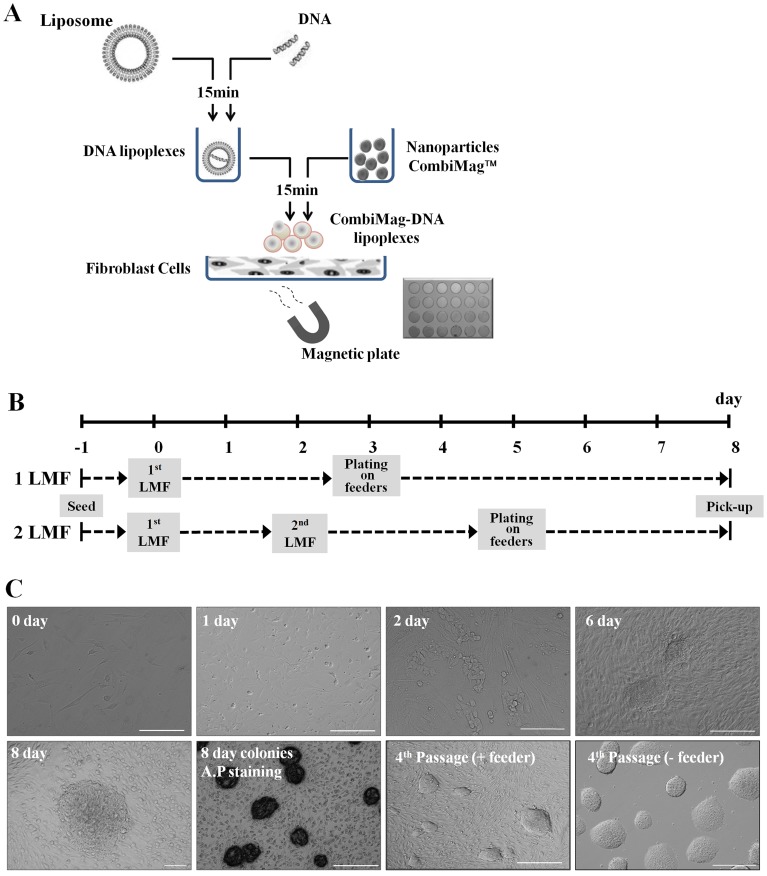
Generation of iPS cells from mouse embryonic fibroblasts (MEF) by liposomal magnetofection (LMF) of two plasmids (pCX-OKS-2A and pCX-cMyc). (A) Methodology of LMF. Two plasmids were mixed with liposomes for 15 min and the resulting DNA lipoplexes were then mixed with CombiMag nanoparticles for a further 15 min. The CombiMag-DNA lipoplexes were added into MEF culture dishes, which were then placed on a magnetic plate. (B) Timeline of LMF-iPS cell production with either one cycle of LMF (1LMF, upper) or two cycles of LMF (2LMF, lower). (C) Morphological changes of MEF cells after LMF and culture of LMF-iPS cells. Two days after LMF, MEF cells were morphologically transformed, and small aggregates were observed at 5–6 days. ES cell-like LMF-iPS cell colonies appeared at 8 days. These colonies exhibited strong alkaline phosphatase (AP) expression. Four passage-cultured feeder-dependent and feeder-independent LMF-iPS cell colonies are shown, with morphological similarity to typical ES cells. Scale bars, 100 µm.

To generate non-viral mouse iPS cells, we attempted LMF using liposomes and CombiMag nanoparticles with iPS genes. As shown in Figure 1A, iPS genes were incubated with liposomes for 15 min and allowed to form DNA lipoplexes. Then, they were incubated with CombiMag nanoparticles for another 15 min. Finally, the prepared CombiMag-DNA lipoplexes were added into MEF cell culture dishes and cultured on a magnetic plate for 15 min. MEF cells were treated with either 1.5 µg or 0.75 µg of the two plasmids (pCX-OKS-2A, OKS; pCX–cMyc, C), and underwent LMF either once or twice. As shown in Figure 1B, irrespective of the treatment conditions, from day 2 post-LMF, morphological changes were apparent in LMF-MEF cells (Figure 1C). Aggregates were clearly formed from 5 to 6 days post-LMF, and the masses grew into ES cell-like colonies at 8 days (Figure 1C). These serial morphological changes, which indicate reprogramming, seemed very similar to those induced by viral infection. Thus, our virus-free LMF method might be a powerful reprogramming method. Okita et al. [16] used the same vector as in the present study but required four transfections to make iPS cells, and iPS cell colonies required 25 days for formation; hence, the reprogramming efficiency was very low. In our results, from four treatment conditions and three replications, 438 (1LMF-o, 120; 1LMF-h, 105; 2LMF-o, 97; 2LMF-h, 116; Table 1) AP-expressing colonies were confirmed at 8 days (Figure 1C). Of these, 15 LMF-iPS cell lines were selected and successfully established (1LMF-o, 3 lines; 1LMF-h, 4 lines; 2LMF-o, 4 lines; 2LMF-h, 4 lines; Table 1). The reprogramming efficiency of each treatment group was 0.032–0.04% based on AP-positive clones, which constituted the input cells (1LMF-o: 0.04%, 1LMF-h: 0.035%, 2LMF-o: 0.032%, 2LMF-h: 0.039%), and there were no differences among the treatment groups. These levels of efficiency were high compared with those of other non-viral systems (0.0001–0.0012%; Okita et al. [16]). In addition, our protocol was very simple and fast, and yielded similar results to those of retroviral vector systems (0.02%, Takahashi and Yamanaka. [1]; 0.05–0.1%, Wernig et al. [2]). All of the established LMF-iPS cell lines were expanded homogenously for over 20 passages and showed morphological similarity to classic D3 ES cells known to form compact, domed, small colonies on STO feeder cells or feeder-free gelatin-coated dishes (Figure 1C).

**Table 1:** Generation of mouse induced pluripotent stem cells by liposomal magnetofection (LMF) under different conditions.

Treatment group*	LMF no.	DNA conc. (ug)	A.P positive clones	Established LMF-iPS clones		Examined LMF-iPS clones	Integration-free LMF-iPS clones
				Total	Name		
**1LMF-o**	**1**	1 (OKS 1, C 0.5)	120	3	1LMF-o-iPS 1	**√**	
					1LMF-o-iPS 2	ND	−
					1LMF-o-iPS 3	ND	−
**1LMF-h**		1/2 (OKS 0.5, C 0.25)	105	4	1LMF-h-iPS 1	**√**	
					1LMF-h-iPS 2	**√**	**√**
					1LMF-h-iPS 3	ND	−
					1LMF-h-iPS 4	ND	−
**2LMF-o**	**2**	1 (OKS 1, C 0.5)	97	4	2LMF-o-iPS 1	**√**	
					2LMF-o-iPS 2	**√**	**√**
					2LMF-o-iPS 3	ND	−
					2LMF-o-iPS 4	ND	−
**2LMF-h**		1/2 (OKS 0.5, C 0.25)	116	4	2LMF-h-iPS 1	**√**	
					2LMF-h-iPS 2	**√**	
					2LMF-h-iPS 3	ND	−
					2LMF-h-iPS 4	ND	−

* 1LMF-o: single liposomal magnetofection with a full concentration of two plasmids (1.5 µg).

1LMF-h: single liposomal magnetofection with a half concentration of two plasmids (0.75 µg).

2LMF-o: double liposomal magnetofection with a full concentration of two plasmids (1.5 µg).

2LMF-h: double liposomal magnetofection with a half concentration of two plasmids (0.75 µg).

**Figure 2:**
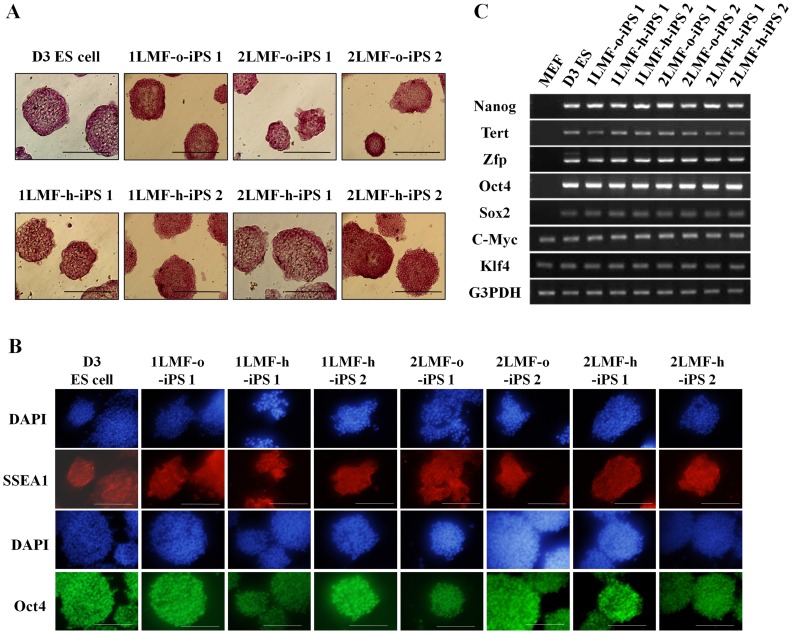
Characterization of LMF-iPS cells: comparison of MEF and mouse D3 ES cells. (A) Alkaline phosphatase (AP) staining. Seven LMF-iPS cell lines examined expressed high levels of AP, similar to D3 ES cells. (B) Immunofluorescence staining of the ES cell-specific markers Oct4 and stage-specific embryonic antigen 1 (SSEA1). Scale bars: 200 µm. (C) mRNA expression of ES cell-specific markers (Nanog, Tert, Zfp, Oct4, and Sox2) and exogenous genes (Kl4 and c-Myc). G3PDH was used as a loading control.

To analyze the iPS cell characteristics, we selected seven LMF-iPS cell lines from four treatment groups (1LMF-o-iPS 1; 1LMF-h-iPS 1, 2; 2LMF-o-iPS 1, 2; 2LMF-h-iPS 1, 2) after more than three passages on gelatin-coated dishes. All seven LMF-iPS cell lines expressed high levels of alkaline phosphatase (Figure 2A) and stained strongly positive for Oct4 and SSEA1 (Figure 2B). In addition, they showed reactivation of ES cell marker genes, including Nanog, Tert, Zfp, Oct4, and Sox2, with similar levels of expression, as seen in D3 ES cells by RT-PCR analysis, whereas MEF cells did not show reactivation of these genes. Also, the expression levels of two exogenous genes (c-Myc and Klf4) were also comparable to those of D3 ES cells (Figure 2C).

### Analysis of Transgene Integration into LMF-iPS Cells

**Figure 3:**
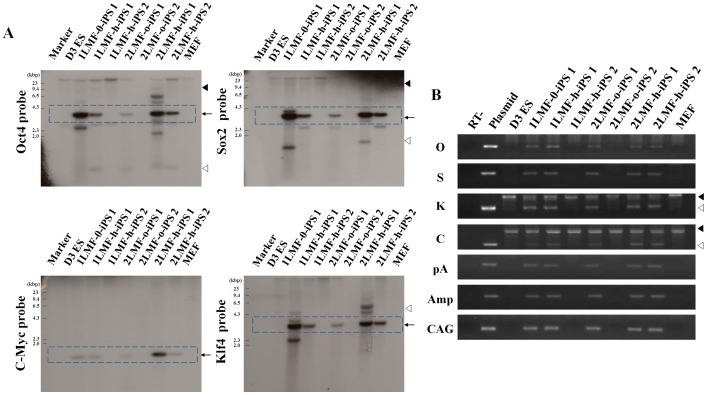
Integration analysis of LMF-iPS cell lines. (A) Southern blot analysis of D3 ES cells, seven LMF-iPS cell lines, and MEF cells using probes against Oct4, Sox2, Klf4, and c-Myc. Genomic DNA (15 µg) was digested with EcoRI. The arrows indicate bands derived from the transgenes. Closed and open arrowheads indicate bands derived from endogenous genes or nonspecific bands integrated into the genomic DNA, respectively. (B) Detection of plasmid integration by PCR. Genomic DNA (100 ng) from D3 ES cells, LMF-iPS cells, and MEFs was amplified by PCR using plasmid-specific primers (amplified regions: O, Oct4; S, Sox2; K, Klf4; C, cMyc; pA, polyadenylation signal and backbone; Amp, ampicillin resistant gene; and CAG, CAG promoter). In the K and C PCR results, closed arrowheads indicate bands derived from endogenous genes and open arrowheads indicate integrated exogenous genes.

To examine the possibility of iPS gene integration, we analyzed seven LMF-iPS cell lines by Southern blot using probes against Oct4, Sox2, c-Myc, and Klf4. In two of the LMF-iPS cell lines (1LMF-h-iPS 2 and 2LMF-o-iPS 2), exogenous genes were not detected, but such genes were detected in the other five LMF-iPS cell lines (1LMF-o-iPS 1, 1LMF-h-iPS 1, 2LMF-o-iPS 1, 2LMF-h-iPS 1, and 2LMF-h-iPS 2). Endogenous genes, however, were present in all seven LMF-iPS cell lines (Figure 3A). In addition, we confirmed this result through genomic DNA PCR analyses. We used seven sets of PCR primers to amplify various parts of the plasmids (Table S3) and confirmed that no amplification of plasmid DNA occurred in two out of seven LMF-iPS cell lines, regardless of LMF number and DNA concentration (Figure 3B). The advantage of a virus-free system is the recovery of integration-free iPS cell clones. This study introduced the LMF method for making iPS cells and demonstrated several merits of this method, including a reasonable level of efficiency and the generation of two integration-free LMF-iPS cell lines from seven LMF-iPS cell lines (28.6%, 2/7). Above all, improved efficiency in the generation of iPS cells, compared with other virus-free methods, is a very attractive point. This improved efficiency was especially evident in the case of the 1LMF-h-iPS 2 cell line, which was generated under LMF conditions using a half dose of DNA and a single LMF treatment. Considering the need for low concentrations of DNA and magnetoparticles, plus other positive factors such as rapid reprogramming and a highly effective protocol, the generation of integration-free iPS cells using LMF might be advantageous compared with using other non-viral systems.

### Pluripotency of LMF-iPS Cells

**Figure 4:**
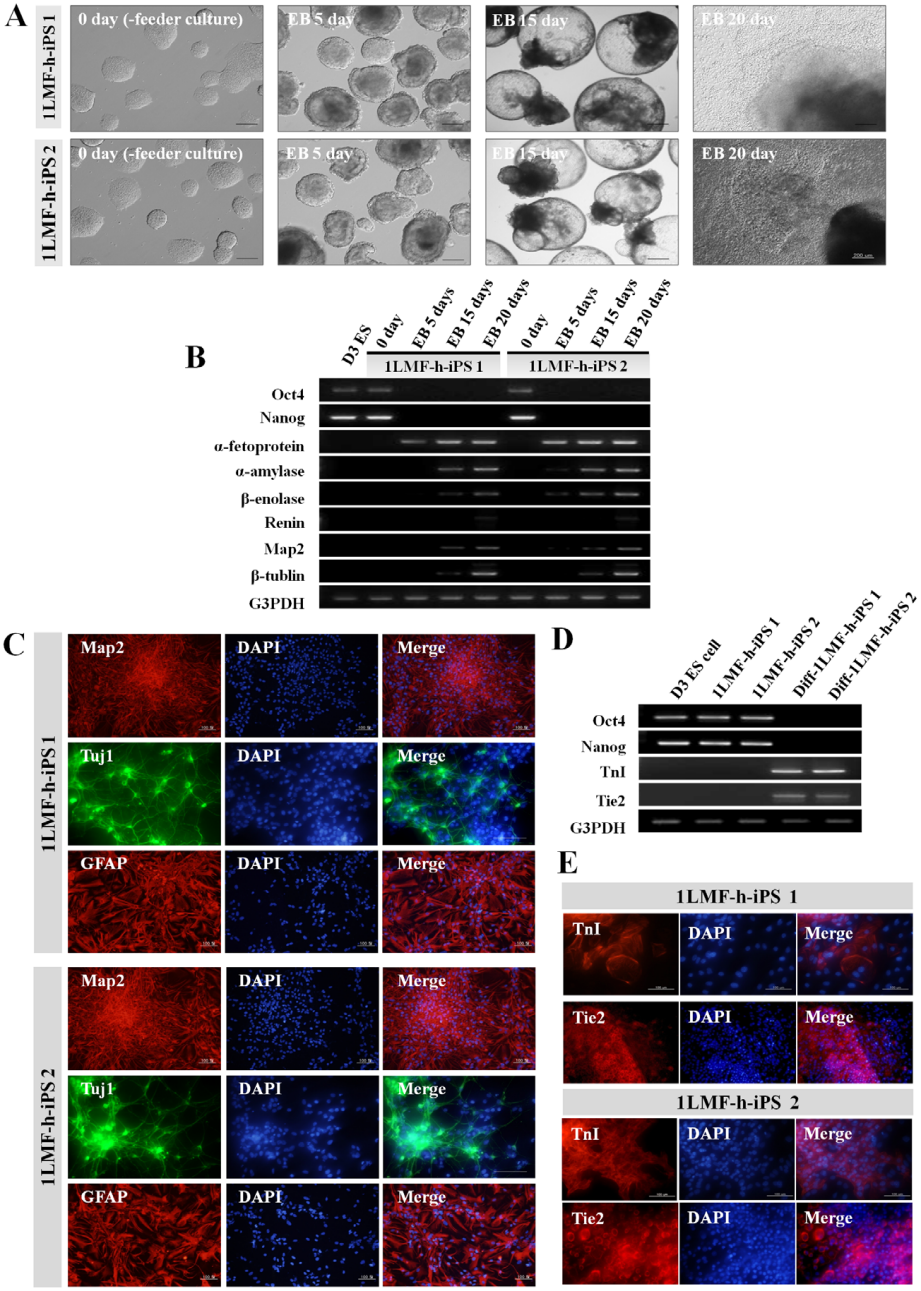
Comparison of *in vitro* differentiation potential between integrated (1LMF-h-iPS1) and integration-free LMF-iPS cell lines (1LMF-h-iPS2). (A) Phase-contrast images of the two LMF-iPS cell lines, according to spontaneous differentiation for 20 days. (B) RT-PCR analysis of the spontaneous differentiation of the two LMF-iPS cell lines into three germ-like layers expressing undifferentiated ES cell markers (Oct4 and Nanog), as well as endodermal (α-fetoprotein and α-amylase), mesodermal (β-enolase and renin), and ectodermal (Map2 and β-tubulin) genes. (C) Immunocytochemistry of neuronal cell differentiation in the two LMF-iPS cell lines into Map2-, Tuj1-, and GFAP-positive cells. (D and E) RT-PCR and immunostaining results showing cardiac (TnI) and endothelial (Tie2) cell differentiation of two LMF-iPS cell lines. Blue nuclear staining is by DAPI.

For comparative analysis of differentiation potential, we selected two LMF-iPS cell lines, transgene-integrated 1LMF-h-iPS 1 cells and integration-free 1LMF-h-iPS 2 cells, from the same 1LMF-h treatment group. To determine spontaneous differentiation potential *in vitro*, embryoid bodies (EBs) from the two LMF-iPS cell lines were allowed to form for 15 days, and attached EBs were cultured for another 5 days (Figure 4A). Then, the cells were analyzed for cell differentiation into three germ layers by RT-PCR. There were no differences in the morphological change between the two LMF-iPS cell lines during *in vitro* differentiation. Also, the expression of endoderm genes (α-fetoprotein and α-amylase), mesoderm genes (β-enolase and renin), and ectoderm genes (Map2 and β-tubulin) increased with differentiation time, whereas expression of Oct4 and Nanog was not strictly detected from EBs after 5 days of culture (Figure 4B).

To verify *in vitro* differentiation potential into specific cell types, we induced the direct differentiation of 1LMF-h-iPS-1 and 1LMF-h-iPS-2 cells. For neuronal cell differentiation, the majority of the 2 LMF-iPS cell lines showed positive immunostaining for neuron-specific microtubule-associated protein 2 (Map2), class III beta-tubulin (Tuj1), and glial fibrillary acidic protein (GFAP) (Figure 4C) after a 16 day differentiation period. Also, *in vitro* cardiac and endothelial cell differentiation, expression of cardiac troponin I (TnI), and expression of endothelial-specific receptor tyrosine kinase (Tie2) were detected in 1MF-h-iPS-1 and 1MF-h-iPS-2 cells by RT-PCR (Figure 4D) and immunocytochemistry (Figure 4E), respectively. In addition, regarding cardiac cell differentiation, beating masses were confirmed in the two LMF-iPS cell lines (Video S1 and S2). These results indicated that there were no differences in *in vitro* differentiation potential according to transgene integration or a lack of integration in accordance with the results of Gonzalez et al. [21].

**Figure 5:**
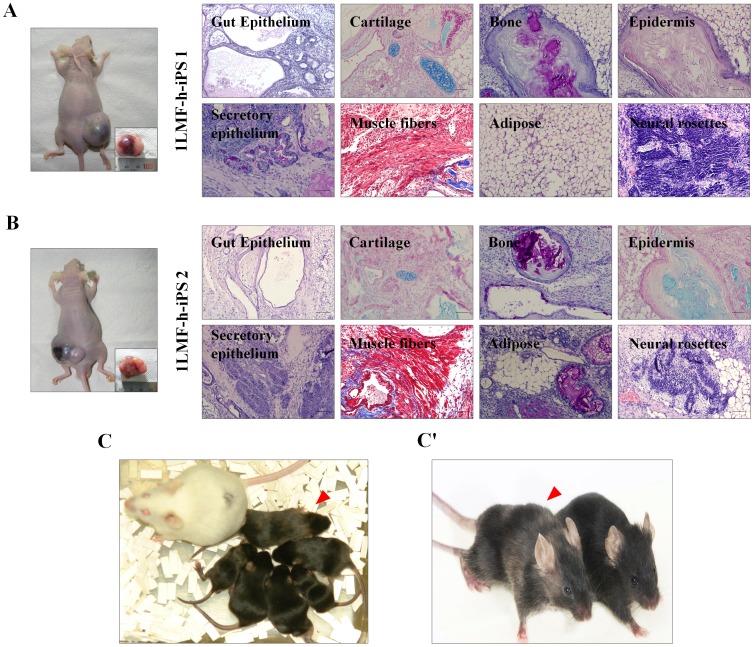
*In vivo* validation of the pluripotency of 1LMF-h-iPS 1 and 1LMF-h-iPS 2 cells. Teratoma formation in mice and tissue sections showing the formation of cells from the three germ layers: endoderm (gut epithelium and secretory epithelium), mesoderm (adipose, bone, cartilage, and striated muscle fibers), and ectoderm (epidermis and neural rosettes) in 1LMF-h-iPS 1 (A) and 1LMF-h-iPS 2 cells (B). (C and C’) Chimeric mouse produced by injecting 1LMF-h-iPS 2 cells into c57BL/6 host blastocysts. The natural coat color of the surrogate strain ICR is white, and the brown hair (red arrowhead) indicates a chimeric mouse (red) that originated from the 1LMF-h-iPS 2 cells.

To further analyze differentiation potential *in vivo*, transgene-integrated 1LMF-h-iPS 1 cells and integration-free 1LMF-h-iPS 2 cells were injected into the femoral muscles of severe combined immunodeficient mice. This resulted in the formation of 2 cm-sized teratomas with cells that had differentiated into three germ layers: endoderm (gut epithelium and secretory epithelium), mesoderm (cartilage, muscle fibers, bone, and adipose) and ectoderm (epidermis and neural rosettes) (Figure 5A and B), as confirmed by eosin and hematoxylin staining. There were no differences in *in vivo* pluripotency between the two LMF-iPS cell lines. Also, when the integration-free CDF1 1LMF-h-iPS 2 cells (brown colored hair) were injected into the two hundred c57BL/6 blastocysts (black colored hair) from the nine surrogates, 36 (36/200, 18.0%) offspring were delivered by cesarean section. In these offspring, seven chimeric mice (7/36, 19.4%), as judged from coat color (black and brown mixed hair), were obtained (Figure 5C and C’). In view of the fact that chimeric development of iPS cells indicates complete reprogramming [8], [22], [2], and the fact that the iPS cell line used for chimeric mouse production in the present study is integration-free, it can be inferred that transgene integration is not required for *in vitro* reprogramming. We therefore conclude that our LMF method has potential for the stable generation of iPS cells.

### Safety of Liposomal Magnetofection in MEF Cells

**Figure 6:**
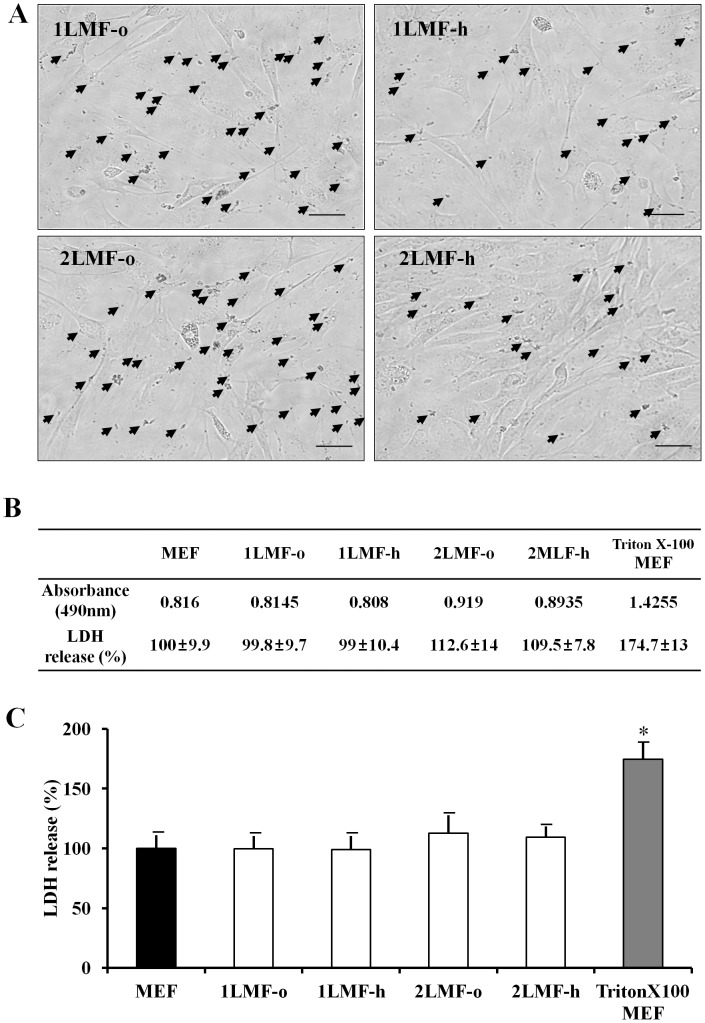
Cytotoxicity determination of MEF cells after LMF. (A) Liposomal magnetofection particles (arrows) in MEF cells at 24 hr post-LMF with either one or two LMF procedures and with different DNA doses (o: 1.5 µg, h: 0.75 µg). (B and C) The colorimetric absorbance values and LDH release percentages for control MEF cells, the four LMF treatment groups (1LMF-o, 1LMF-h, 2LMF-o, and 2LMF-h) at 24 hr post-LMF, and triton X-100–exposed MEF cells.

To assess cytotoxicity in MEF cells according to single or double LMF treatment with different DNA concentrations, we measured lactate dehydrogenase (LDH) activity in the cytoplasm of intact cells at 24 h post-LMF using the CytoTox 96® Non-Radioactive Cytotoxic Assay. Although a number of LMF particles were observed in each treatment group at 24 h post-LMF (Figure 6A), after that time period, no detrimental effects were observed in any of the treatment groups. Measurement of LDH release in control MEF cells, the four LMF treatment groups at 24 h post LMF, and Triton X-100–exposed MEF cells (Figure 6B and C) indicated that the LMF treatment groups (1LMF-o: 99.8±9.7%; 1LMF-h: 99±10.4%; 2LMF-o: 109.5±7.8%; 2LMF-h: 112.6±14%) were not different from the control MEF cells (100±9.9%) in terms of LDH release, in contrast to the Triton X-100–treated MEF cells (174.7±13%). However, there were slight increases in LDH activities according to LMF number and DNA concentration (1LMF-h<1LMF-o<2LMF-h<2LMF-o), and these results were similar to those of Krotz et al. [23] and Prijic et al. [24].

### Transmission Electron Microscopic Analysis of Liposomal Magnetofected MEF Cells

**Figure 7:**
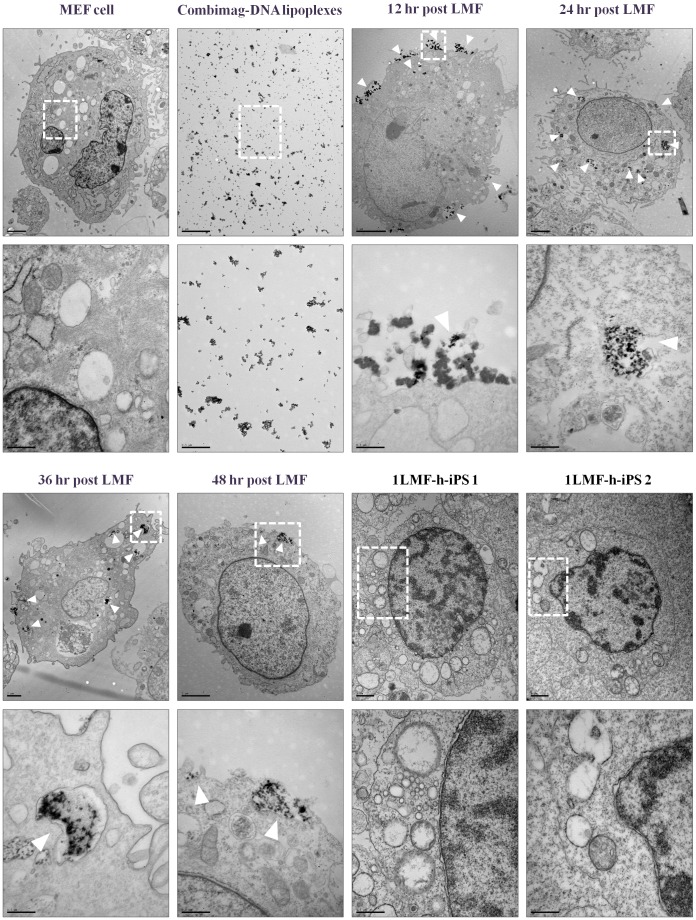
Transmission electron microscopic analysis of MEFs, CombiMag-DNA lipoplexes, LMF-treated MEF cells at 12 hr intervals until 48 hrs, and two LMF-iPS cell lines. The lower photos represent magnifications of the white-dotted square in each upper photo, and the white arrowheads indicate the presence of liposomal magnetofection particles in LMF-MEF cells. Upper: low magnification, x6,000–8,000; lower: high magnification, x50,000.

To trace the liposomal magnetofection particle location in MEF cells after LMF, we investigated the position of CombiMag-DNA lipoplexes by TEM analysis. Single CombiMag nanoparticles were approximately 2 to 10 nm in diameter. TEM analysis indicated that the cellular uptake of CombiMag-DNA lipoplexes was mediated by endocytosis. Unlike control MEF cells, CombiMag-DNA lipoplexes mostly accumulated at the cell surface (white arrowheads) at 12 h post-LMF, and numerous CombiMag-DNA lipoplexes were observed in the cytoplasm of MEF cells at 24 h post-LMF. However, from 36 h post-LMF, the CombiMag-DNA lipoplex numbers were markedly declined and they had moved toward the cytoplasmic membrane of the MEF cells. Finally, at 48 h post-LMF, the complexes were barely visible in the MEF cells. After eight passages of cultured, transgene-integrated 1LMF-h-iPS 1 cells and integration-free 1LMF-h-iPS 2 cells, no LMF particles were present in the cytoplasm (Figure 7). This appearance was consistent with the mRNA expression levels indicated in Figure S1.

## Discussion

The generation of iPS cells is a powerful tool, considering the potential usefulness of such cells in regenerative medicine [25]. In the present study, we introduced the liposomal magnetofection (LMF) method in order to enhance the reprogramming efficiency of somatic cells using non-viral vectors. Using simple mixing of polyelectrolyte-coated magnetic particles and gene vectors at an appropriate ratio in a salt-containing medium, this method resulted in the rapid and efficient reprogramming of iPS cells at a level similar to that of viral vector systems, even at low vector doses. Through optimization of the procedures, even under the least toxic conditions (a half dose of vector and a single repetition of LMF), we generated an integration-free iPS cell line derived from MEF cells, and these results were confirmed in additional experiments (Figure S2).

On the other hand, a high content of magnetic particles and repeated LMF cycles might lead to a drop in transfection efficiency and stability, since the increased magnetic field power and vector dose may lead to toxicity [26]. In the present study, we optimized the LMF protocol and found that increasing the LMF repetition number does not necessarily give better results. The reprogramming efficiencies of the 2LMF treatment groups (0.032–0.039%) were in fact slightly lower than those of the 1LMF treatment groups (0.035–0.040%). Meanwhile, cytotoxic LDH release (9.72–13.7%) in the 2LMF treatment groups was higher than that in the 1LMF treatment groups. This indicates that a half dose of plasmid and a single repetition of LMF is sufficient to induce the expression level of factors necessary for reprogramming and may be advantageous in terms of a lower level of cytotoxic effects. However, our optimized DNA quantity was much lower (1/3–1/8) than the recommended quantity. Unlike other non-viral iPS cell generation methods [16], [17], [21], LMF is a simple, effective (>10–30 fold), and reproducible transfection method, and it can be an ideal alternative to viral vector systems without the obstacles to cell therapy commonly seen in viral vector systems, such as oncogenesis and teratoma formation. In our results, signs of reprogramming appeared from 2 days post-LMF, and iPS-like colonies could be selected and subcultured on the STO feeder layer at 8 days after a single LMF. In contrast, the above-mentioned non-viral reprogramming methods presented iPS-like colonies that could only be detected at 3 to 5 weeks (23–35 days), even after multiple transfections (2 to 4).

In the present study, our LMF-iPS cells presented very similar cellular characteristics to typical ES cells, with comparable morphology, surface marker expression, embryoid body formation, teratoma formation, direct differentiation into neural, cardiac, and endothelial cells, and chimeric mouse production, as in other reports [7], [8], [27]. In the generation of iPS cells, the roles of the genes used for reprogramming are crucial, as the success of the technique depends on the levels and patterns of expression of the factors used in transfection. In the present study, we confirmed that core pluripotent gene expression levels were stable in all seven iPS cell lines examined. Also, it is known that during differentiation, in iPS cells, the transcript levels of integrated transgenes that activate the transcriptional regulatory circuit of pluripotent cells are regulated by different mechanisms from those of endogenous genes. The degree of silencing of the integrated genes in particular has an effect on differentiation efficiency [28]. Considering this, we compared the differentiation potential of integrated- and non-integrated LMF-iPS cells. Both integrated and non-integrated LMF-iPS cells formed canonical embryoid body (EB) structures during the same period, and reprogramming gene expression was critically decreased at the differentiation stage. Although the present study found that the induction of endoderm gene expression of non-integrated 1LMF-h-iPS2 cells was slightly faster than that of integrated 1LMF-h-iPS1 cells in RT-PCR analysis, it found similar differences in other iPS cell lines, and thus the differences in differentiation characteristics between integrated and non-integrated LMF-iPS cells may be due to iPS cell clone differences. However, histological examination indicated no apparent differences between the two different kinds of iPS cell lines. In addition, we produced chimeras derived from non-integrated 1LMF-h-iPS2 cells, and all seven chimeras survived for 6 weeks and no somatic damage or tumors were discovered upon necropsy. These results suggested that LMF-iPS cells were completely reprogrammed and had obtained perfect pluripotent differentiation capacities as ES cells.

The generation of non-toxic and efficient patient-specific iPS cell lines is a critical point for future stem cell therapies. Until now, there have been no reports on the location of MF particles in host cells according to time post-transfection. In this study, we present detailed evidence of the disappearance of these particles using TEM analysis. As shown in our results, uptake of LMF particles into the cell is mediated by endocytosis, beginning with invagination of the plasma membrane [29], [24]. In addition, biodegradable LMF particles made of iron oxide have been reported that do not influence any cellular functions [30]. The present study indicated no detrimental morphological changes after LMF, and this fact was confirmed by LDH analysis. Also, this study indicated that the increase and decrease in LMF particle numbers in cells over time was considerably related to the simultaneous expression levels of four reprogramming factors. This expression was enhanced starting at 12 h post-LMF, reached a peak at 24 h post-LMF, and then gradually diminished but remained detectable at 48 h post-LMF, according to real-time PCR.

In conclusion, this newly introduced LMF method is a simple, effective, safe, and reproducible method for the generation of virus-free iPS cells. This non-toxic LMF technique may be an ideal tool for the production of integration-free iPS cell lines, avoiding the risk of transgene reactivation leading to tumor formation. Finally, this approach represents a potentially important step toward the derivation of clinically relevant human iPS cells.

## Materials and Methods

Unless stated otherwise, all chemicals were purchased from Sigma Chemical Company (St Louis, MO, USA).

### Cell Culture

For mouse embryonic fibroblast (MEF) isolation, uteri were isolated from 13.5 day-pregnant CDF1 mice and washed with phosphate buffered saline (PBS). The heads and visceral tissues of the fetuses were removed, and the remaining bodies were washed with fresh PBS, transferred into TrypLE solution (Gibco, Grand Island, NY, USA), and incubated for 30 min. After incubation, MEF cell culture medium, Dulbecco’s Modified Eagle’s Medium (DMEM, Gibco) containing 10% defined fetal bovine serum (FBS, Hyclone, Logan, UT, USA), and 1% penicillin/streptomycin (Gibco) were added and pipetted up and down to dissociate the cells. The dissociated cells were placed in 100 mm culture dishes and cultured in a 5% CO_2_ incubator at 37°C. The cells were grown to confluence on the culture dishes, dissociated using TrypLE solution, and then cryopreserved in liquid nitrogen using MEF cell culture medium containing 10% DMSO. STO cells (a mouse embryonic fibroblast cell line) were purchased from the American Type Culture Collection (ATCC, USA) and cultured in DMEM supplemented with 10% defined FBS, 0.1 mM nonessential amino acids (NEAA), 0.1 mM sodium pyruvate, 100 mM β-mercaptoethanol (Gibco), and 1% penicillin/streptomycin (Gibco).

### Plasmid

The pCX-OKS-2A (OKS, plasmid vector expressing Oct4, Klf4, and Sox2) and pCX–cMyc (C, plasmid vector expressing cMyc) were purchased from Addgene. The two plasmids were mixed at a ratio of 2 OKS per 1 C, at two doses: a full-dose, which was a total of 1.5 µg (1.0 µg of OKS and 0.5 µg of C); and a half-dose, which was a total of 0.75 µg (0.5 µg OKS and 0.25 µg C).

### Liposomal Magnetofection (LMF) and iPS Cell Generation

MEF cells were thawed and cultured in MEF cell culture medium. At 80% confluence, MEF cells were dissociated using TrypLE solution and then seeded at 1×10^5^ cells per well of gelatin-coated, 6-well plates, one day before LMF. Figure 1A shows the methodology of liposomal magnetofection (LMF) used in the generation of iPS cells. First, 94.5 or 95.0 µl of DMEM containing 1.5 µg (full-dose) or 0.75 µg (half-dose) of plasmids was mixed with 4.5 ul of Fugene 6 transfection reagent (Roche, Indianapolis, IN) by vortex and incubated at RT for 15 min. Then, 1 µl or 0.5 µl CombiMag (Chemicell GmbH, Berlin, Germany) was added and mixed by vigorous pipetting. The DNA lipoplexes and CombiMag were incubated at RT for 15 min. At the same time, the culture medium was replaced with FBS and antibiotic-free DMEM. The CombiMag-DNA lipoplexes were then added into each well, for a total LMF volume per well of 1 mL (DMEM 900 µl + CombiMag-DNA lipoplexes 100 µl). The 6-well plates were then placed upon a magnetic plate in a 5% CO_2_ incubator at 37°C for 15 min. The magnetic plate was then removed and the medium was exchanged with ES cell culture medium [DMEM containing 20% defined FBS, 0.1 mM NEAA, 0.1 mM sodium pyruvate, 100 mM β-mercaptoethanol, and 10^3^ units/ml leukemia-inhibitory factor (LIF) (ESGRO: Chemicon International, Middlesex, UK)]. On day 3, the liposomal magnetofected cells were harvested and plated onto STO feeder cells. On day 7 to 8, ES cell-like colonies were picked up for expansion, and then they were cultured with or without STO feeder cells in ES cell culture medium. In the two-cycle LMF condition (2LMF), the LMF procedure was repeated 2 days after the first LMF; the medium was exchanged again with FBS and antibiotic-free DMEM and then cells were subjected to liposomal magnetofection once more, using the same method as with the one-dose condition (1LMF) (Figure 1A and B).

### Alkaline Phosphatase Staining and Immunocytochemistry

Alkaline phosphatase (AP) staining was performed according to the manufacturer’s protocol with the Alkaline Phosphatase Detection Kit (Millipore, Billerica, MA, USA). For immunocytochemistry analysis, cells were fixed in 4% paraformaldehyde for 20 min at 4°C. The following antibodies were used: Oct4 (1∶250; Santa Cruz Biotechnology, Santa Cruz, CA, USA), SSEA1 (1∶50; Santa Cruz), Map2 (1∶1,000; Chemicon), Tuj1 (1∶1,000; Chemicon), GFAP (1∶1,000; Chemicon), TnI (1∶100; Santa Cruz), and Tie2 (1∶200; eBioscience). The secondary antibodies used were goat anti-mouse tetramethylrhodamine isothiocyanate (TRITC, 1∶200, Jackson Laboratories, West Grove, PA) and goat anti-mouse fluorescein isothiocyanate (FITC, 1∶200, Jackson Laboratories). Nuclei were stained with 5 µg/ml of 4′-6-diamidino-2-penylindole (DAPI, Invitrogen, Grand Island, NY, USA).

### RT-PCR

Total RNA was isolated from MEF cells, D3 ES cells, LMF-iPS cell lines and in vitro differentiated LMF-iPS cell lines using the TRIzol reagent. Total RNA was treated with DNase I (Invitrogen) to remove any potential contamination with genomic DNA. Five micrograms of DNase I-treated total RNA were converted to cDNA using Improm II reverse transcriptase (Promega, Wisconsin, USA) and Oligo dT primers (Bioneer, Daejeon, Korea). PCR was performed with EX Taq (Takara, Shiga, Japan). PCR products were resolved on (1%) agarose gels and visualized by ethidium bromide staining. Images were taken using the Bio-Rad Gel Documentation System. Amplification of specific genes was done using the primers described in Table S1 and Table S2.

### Southern Blotting

D3 ES cells, LMF-iPS cells, and MEF cells were suspended in cell lysis buffer (50 mM Tris-HCL, pH 8.0, 400 mM NaCl, 100 mM EDTA, 0.5% sodium dodecylsulfate) supplemented with Proteinase K (50 µg/ml, Takara). After overnight incubation at 55°C, high molecular weight DNA was extracted with a Genomic DNA Prep Kit (SolGent, Daejeon, Korea). Fragments resulting from overnight 100 Unit/µl EcoRI (Enzynomics, Daejeon, Korea) digestion of 20 µg of genomic DNA were separated on a 0.7% agarose gel, transferred to a nylon membrane (Amersham, Buckinghamshire, UK) in 10X SSC buffer (3 M sodium chloride and 0.3 M sodium citrate), and hybridized with ^32^P random primer-labeled probes (30 ng) in solution containing 5X SSPE. The sequences of the primers used to amplify the probe are supplied in Table S3.

### PCR

Genomic DNA was extracted from D3 ES cells, LMF-iPS cells, and MEF cells using the Genomic DNA Prep Kit. The genomic DNAs (100 ng each) and the plasmid DNA mixture (100 fg of OKS and C) were analyzed by PCR amplification of integration fragments, as shown in Table S4. PCR was performed with EX Taq.

### *In vitro* Differentiation

Before differentiation was initiated, LMF-iPS cells were subcultured up to three times without feeder cells on 60 mm culture dishes coated with 0.1% gelatin to eliminate contaminating STO feeder cells. For spontaneous differentiation, integrated 1LMF-h-iPS 1 and non-integrated 1LMF-h-iPS 2 cells were dissociated using TrypLE solution and transferred to low-attachment, 100 mm petri dishes (Falcon, Franklin Lakes, NJ, USA) in ES cell culture medium without LIF and cultured for 15 days. Cystic embryoid bodies (EBs) were then plated on 0.1% gelatin-coated culture dishes and cultured in the same medium for an additional 5 days. During differentiation, the medium was replaced every 2–3 days.

For direct neuronal cell differentiation, day 8 EBs of 1LMF-h-iPS 1 and 1LMF-h-iPS 2 cells were dissociated with TrypLE solution and single cells were cultured onto 0.1% gelatin-coated, 4-well plates in N2 medium, consisting of DMEM/F12 supplemented with 0.01% BSA, 20 nM progesterone, 100 µM putrescine, 25 µg/ml insulin, 50 µg/ml transferrin, and 30 nM sodium selenite. Immunocytochemistry was performed after an additional 8 days of culture. To induce cardiac cell differentiation, day 4 EBs of 1LMF-h-iPS 1 and 1LMF-h-iPS 2 cells were transferred to Microvascular Endothelial Cell Basal Medium (EBM-2, Lonza, Walkersville, USA) and cultured for four additional days. On day 8, cystic EBs were plated onto 0.1% gelatin-coated tissue culture dishes and allowed to differentiate in EBM-2 medium for 12 days. The beating EBs were then subjected to immunocytochemistry. For endothelial cell differentiation, day 4 EBs from 1LMF-h-iPS 1 and 1LMF-h-iPS 2 cells were plated onto gelatin-coated, 4-well dishes and cultured in EBM-2 medium with 100 µM ascorbic acid for 2 weeks.

### Teratoma Formation

Cultured 1LMF-h-iPS 1 or 1LMF-h-iPS 2 cells passaged 8–12 times (1×10^7^ cells/mL) were injected under the femoral region or back of recipient Balb/c nude mice. Six to eight weeks later, tumors were surgically dissected from the mice. Tumors were weighed, fixed in PBS containing 4% formaldehyde, and embedded in paraffin. Tissue sections were cut and stained with hematoxylin and eosin.

### Generation of Chimeras

Four- to five-week-old female mice (c57BL/6) were induced to superovulation (7.5 I.U. pregnant mare’s serum gonadotropin (PMSG) administration followed after 48 hr by 7.5 I.U. human chorionic gonadotropin (hCG) administration via intraperitoneal injection and mated with c57BL/6 male mice. Blastocysts were collected at day 3.5 after vaginal plug check and flushed in M2 medium. Blastocysts were then extensively washed in M2 medium and cultured in M16 medium containing 10% FBS until cell injection. Integration-free 1LMF-h-iPS 2 cells (20 cells) were injected into each single blastocyst using a micromanipulation pipette in M2 medium, and then the 1LMF-h-iPS 2 cell-injected embryos were cultured overnight in M16 medium at 37°C, 5% CO_2_ in air. The following day, the LMF-iPS cell-injected blastocysts were transplanted into 2.5 days-post-coitus (dpc) pseudopregnant ICR recipient females. The chimeric mice were delivered by cesarean section on 20–21 dpc.

### Cytotoxicity Assay

MEF cells were seeded on gelatin-coated 96-well plates in MEF culture medium and allowed to grow for 24 hr. In the four LMF treatment groups, defined by the number of LMF cycles [once (1LMF) or twice (2LMF)] and/or dose of plasmid DNA [1.5 µg (full dose) or 0.75 µg (half dose)], lactate dehydrogenase (LDH) release was indirectly measured at 24 hr post-LMF using an ELISA plate reader. LDH activity was examined according to the manufacturer’s protocol with the CytoTox 96® Non-Radioactive Cytotoxicity Assay Kit (Promega). Also, the LDH activity of non-treated MEF cells (negative control) or damaged MEF cells exposed to 1% Triton X-100 for 10 min (positive control) was examined.

### Transmission Electron Microscopy (TEM)

To trace LMF particle deposition in the cytoplasm of MEF cells, MEF cells were harvested at the appointed time (12 hr, 24 hr, 36 hr, 48 hr) post-LMF using TrypLE solution and were fixed in a mixture of 2% (w/v) paraformaldehyde and 2% (v/v) glutaraldehyde in 0.05 M sodium cacodylate buffer, pH 7.2, for 2 hr at 4°C. Post-fixation was carried out in 1% osmium tetroxide (OsO4) in 0.05 M sodium cacodylate buffer for 2 hr, followed by dehydration in increasing concentrations of ethanol and embedding in Spurr’s resin. Ultrathin sections (60 nm) were cut with an ultramicrotome (MT-X, Tucson, AZ, USA) and examined with a transmission electron microscope (LIBRA 120, Carl Zeiss, Germany). Also, non-treated MEF cells, CombiMag-DNA lipoplexes, 1LMF-h-iPS 1 cells, and 1LMF-h-iPS 2 cells were analyzed by TEM.

### Real-time PCR

Comparative real-time PCR was performed using a Chromo 4 (BIO-RAD) and DyNAmo HS SYBR Green qPCR kit (Finnzyme Oy, Espoo, Finland), according to the manufacturer’s instructions, with the primers described in Table S5.

## Supporting Information

Figure S1 **The relative expression of introduced genes in liposomal magnetofected MEF cells according to incubation time (0 h, 6 h, 12 h, 24 h, 36 h, and 48 h).** The value of Oct4 expression at 0 h post-LMF was set to 1.
(TIF)

Figure S2 **Detection of plasmid integration in additional iPS cell lines by Southern blot.** Genomic DNA (15 µg) was extracted from D3 ES cells, MEF cells, and eight iPS cell lines (1LMF-o-iPS 2, 1LMF-o-iPS 3, 1LMF-h-iPS 3, 1LMF-h-iPS 4, 2LMF-o-iPS 3, 2LMF-o-iPS 4, 2LMF-h-iPS 3, and 2LMF-h-iPS 4) and was digested with EcoR I. The arrows indicate bands derived from the transgenes.
(TIF)

Table S1 **Primer sequences to detect ES markers.**
(DOCX)

Table S2 **Gene-specific primers for three germ layers for RT-PCR.**
(DOCX)

Table S3 **Primers for probe generation.**
(DOCX)

Table S4 **PCR Primers to detect integration of transgenes.**
(DOCX)

Table S5 **Primers used for real-time PCR.**
(DOCX)

Video S1 **Cardiac beating of 1LMF-h-iPS1 cell mass.**
(WMV)

Video S2 **Cardiac beating of 1LMF-h-iPS2 cell mass.**
(WMV)
