# Supplementary figures and images for: Efficient Generation of Virus-Free iPS Cells Using Liposomal Magnetofection
Source: PLoS One. 2012 Sep 25;7(9):e45812. doi: 10.1371/journal.pone.0045812 (PMC3458059; doi:10.1371/journal.pone.0045812)

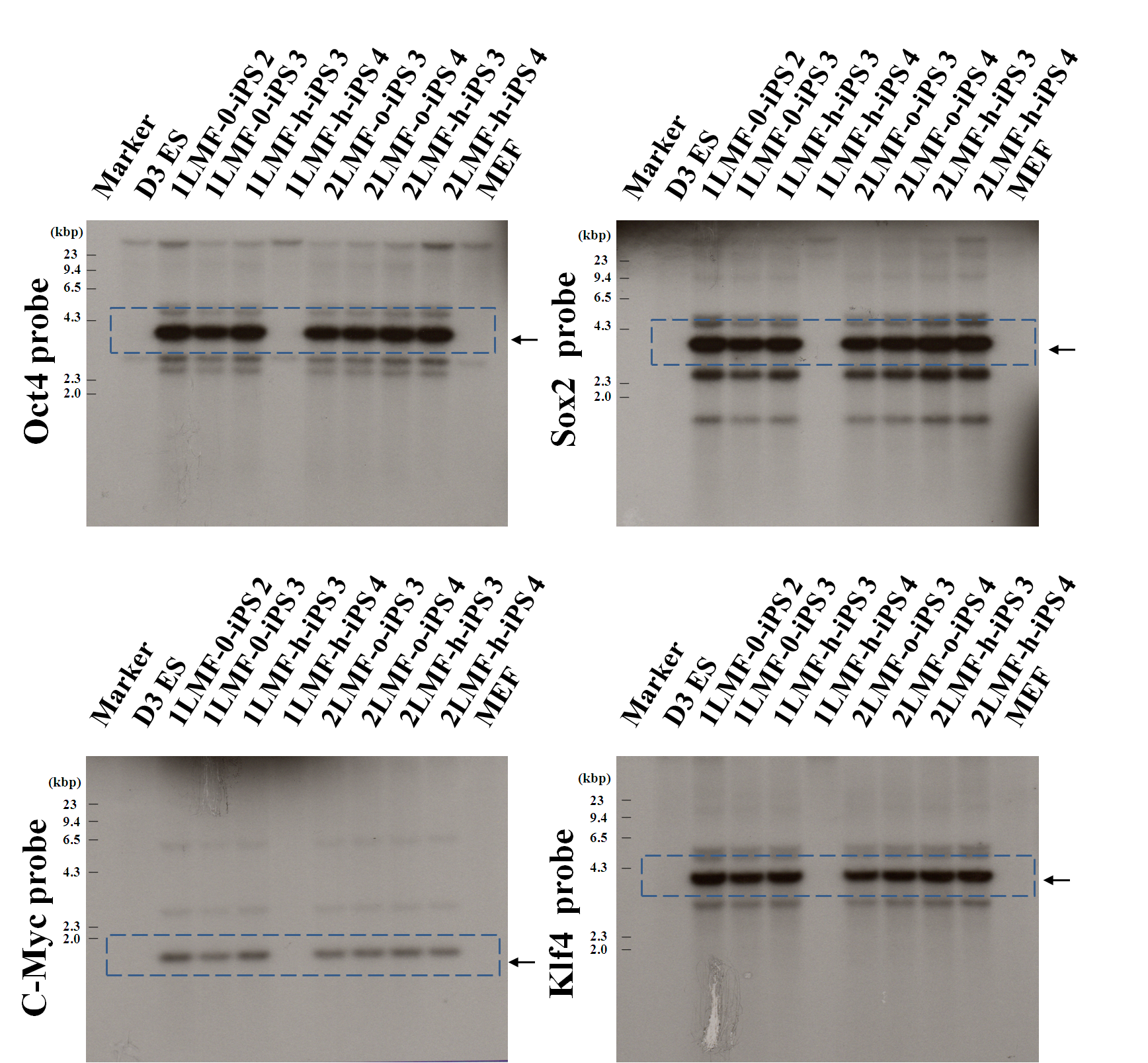

Supplement: Figure S2 — Detection of plasmid integration in additional iPS cell lines by Southern blot. Genomic DNA (15 µg) was extracted from D3 ES cells, MEF cells, and eight iPS cell lines (1LMF-o-iPS 2, 1LMF-o-iPS 3, 1LMF-h-iPS 3, 1LMF-h-iPS 4, 2LMF-o-iPS 3, 2LMF-o-iPS 4, 2LMF-h-iPS 3, and 2LMF-h-iPS 4) and was digested with EcoR I. The arrows indicate bands derived from the transgenes. (TIF) [file pone.0045812.s002.tif]
