# Supplementary material for: Efficient Generation of Virus-Free iPS Cells Using Liposomal Magnetofection
Source: PLoS One. 2012 Sep 25;7(9):e45812. doi: 10.1371/journal.pone.0045812 (PMC3458059; doi:10.1371/journal.pone.0045812)
Supplement: Table S1 — Primer sequences to detect ES markers. (DOCX) [file pone.0045812.s003.docx]

**Supporting Information Table S1.** Primer sequences to detect ES markers.

| **Gene** | **Forward Primer (5’ to 3’)** | **Reverse Primer (5’ to 3’)** |
| --- | --- | --- |
| **Nanog (NM_028016)** | TGAGATGCTCTGCACAGAGG | CAGATGCGTTCACCAGATAG |
| **Tert (NM_009354)** | TGGACACATACGTGGTACAG | GCATGCTGAAGAGAGTCTTG |
| **Zfp (NM_022409)** | CGACACCGACATTGAGATGC | GCAACTTCCAAGGACTAGTG |
| **Oct4 (NM_013633)** | CTAGAGAAGGATGTGGTTCG | TCAGGAAAAGGGACTGAGTA |
| **Sox2 (NM_011443)** | GGAGTGGAAACTTTTGTCC | GGGAAGCGTGTACTTATCCT |
| **c-Myc (NM_010849)** | AGGAAGAAATTGATGTGGTG | CTGGATAGTCCTTCCTTGTG |
| **Klf4 (NM_010637)** | AACCTTACCACTGTGACTGG | AAAAGTGCCTCTTCATGTGT |
| **G3PDH (NM_008084)** | CCCATGTTTGTGATGGGTGT | CCTTCCACAATGCCAAAGTT |
