# Supplementary material for: Efficient Generation of Virus-Free iPS Cells Using Liposomal Magnetofection
Source: PLoS One. 2012 Sep 25;7(9):e45812. doi: 10.1371/journal.pone.0045812 (PMC3458059; doi:10.1371/journal.pone.0045812)
Supplement: Table S2 — Gene-specific primers for three germ layers for RT-PCR. (DOCX) [file pone.0045812.s004.docx]

**Supporting Information Table S2.** Gene-specific primers for three germ layers for RT-PCR**.**

| **Gene** | **Forward Primer (5’ to 3’)** | **Reverse Primer (5’ to 3’)** |
| --- | --- | --- |
| **α-fetoprotein (NM_007423.3)** | GGCAACAACCATTATTAAGC | GCAATTCTTCTTCCAGATTG |
| **α-amylase (NM_007446.1)** | GGTCTGGAAATGAAGATGAA | CTTGGAAAATGAAAGGTCTG |
| **β-enolase (NM_007933.2)** | CTGTGGAACACATCAACAAG | CTCATTGTTCTCCAGGATGT |
| **renin (NM_031192.2)** | GGGCTACACAGCTCTTAGAA | GTAGTGGATGGTGAAGTCGT |
| **Map2 (NM_008632)** | AGTAGTCACAGCTGAAGCTG | GAAGGTGGCAGATTAGCTGT |
| **β-tubulin (NM_023279)** | AAGGTGCGTGAGGAGTACC | TGTGTTCTCCACTAGCTGGT |
| **TnI (NM_009406)** | ACGTGGAAGCAAAAGTCACC | CCTTCTTCACCTGCTTGAGG |
| **Tie2 (NM_013690)** | ACGGACCAGACTGTAAGCTC | CTCTATCTGTGGAGTCATCC |
| **G3PDH (NM_008084)** | GTCGTGGAGTCTACTGGTGT | GTCATCATACTTGGCAGGTT |
