# Supplementary material for: Efficient Generation of Virus-Free iPS Cells Using Liposomal Magnetofection
Source: PLoS One. 2012 Sep 25;7(9):e45812. doi: 10.1371/journal.pone.0045812 (PMC3458059; doi:10.1371/journal.pone.0045812)
Supplement: Table S4 — PCR Primers to detect integration of transgenes. (DOCX) [file pone.0045812.s006.docx]

**Supporting Information Table S4.** PCR Primers to detect integration of transgenes.

| **Gene** | **Forward Primer (5’ to 3’)** | **Reverse Primer (5’ to 3’)** |
| --- | --- | --- |
| **O** | GATCACTCACATCGCCAATC | CTGGGAAAGGTGTCCTGTAGCC |
| **S** | CCTTACACATGAAGAGGCACTTT | CAGCTCCGTCTCCATCATGTTAT |
| **K** | GCGGGAAGGGAGAAGACACTGCGTC | TAGGAGGGCCGGGTTGTTACTGCT |
| **C** | ACACTCCCCCAACACCAGGACGTTT | GAGATGAGCCCGACTCCGACCTCTT |
|  | GCTCGCCCAAATCCTGTACCTCGTCCGA |  |
| **pA** | TGGCGTAATCATGGTCATAG | GCAACGCAATTAATGTGAGTTAG |
| **Amp** | AGTTGCCTGACTCCCCGTCGTG | GGAGCCGGTGAGCGTGGGTC |
| **CAG** | GAAAAGTGCCACCTGGTCGACATT | GGGCCATTTACCGTAAGTTATGTA |
