# Supplementary material for: Efficient Generation of Virus-Free iPS Cells Using Liposomal Magnetofection
Source: PLoS One. 2012 Sep 25;7(9):e45812. doi: 10.1371/journal.pone.0045812 (PMC3458059; doi:10.1371/journal.pone.0045812)
Supplement: Table S5 — Primers used for real-time PCR. (DOCX) [file pone.0045812.s007.docx]

**Supporting Information Table S5.** Primers used for real-time PCR.

| **Gene** | **Forward Primer (5’ to 3’)** | **Reverse Primer (5’ to 3’)** |
| --- | --- | --- |
| **Oct4** | CTAGAGAAGGATGTGGTTCG | TCAGGAAAAGGGACTGAGTA |
| **Sox2** | GGAGTGGAAACTTTTGTCC | GGGAAGCGTGTACTTATCCT |
| **Klf4** | AACCTTACCACTGTGACTGG | AAAAGTGCCTCTTCATGTGT |
| **c-Myc** | AGGAAGAAATTGATGTGGTG | CTGGATAGTCCTTCCTTGTG |
